# Environmental stewardship and robotic surgery: innovation without compromise

**DOI:** 10.1007/s11701-026-03419-0

**Published:** 2026-04-21

**Authors:** Mathew O. Jacob, Gavin J. Carmichael, Noor Naqeeb, Joshua G. Kovoor, Kingsley W. Faulkner

**Affiliations:** 1 Grampians Health, Ballarat, VIC Australia; 2 Grampians Research Initiative (GRIT), Ballarat, VIC Australia; 3 Adelaide University, Adelaide, South Australia Australia; 4 Doctors for the Environment, Melbourne, Australia

**Keywords:** Robotic surgical procedures, Minimally invasive surgical procedures, Environmental policy, Carbon footprint, Sustainability, Medical waste, Greenhouse gases

## Abstract

Robotic surgery represents a major paradigm shift in surgical care, delivering significant improvements in precision, ergonomics, and postoperative patient outcomes. However, the rapid global proliferation of these systems necessitates a critical evaluation of their environmental sustainability. The healthcare sector, particularly within hospital operating theatres, is already a highly resource-intensive environment contributing substantially to national greenhouse gas emissions. The integration of robotic platforms may further exacerbate this ecological footprint through energy-intensive console and robotic arm operations, high consumption of single-use instruments, complex waste streams, and significant upstream manufacturing emissions. Recent life-cycle assessments indicate that robotic systems draw approximately 3.5 kW during operation, generating roughly 4 kg of CO₂ per hour. This is markedly higher than the 0.6 kW and 1 kg of CO₂ per hour associated with conventional laparoscopic equipment. This commentary piece aims to highlight these environmental realities for surgeons and key stakeholders. We do not advocate for the abandonment of robotic surgery, as its clinical benefits remain high. Rather, we issue a call for responsible environmental stewardship and innovation toward ecologically sustainable robotic platforms and components that maintain clinical excellence while mitigating planetary harm.

## Introduction

Robotic surgery is a major innovation in surgical care; improving intraoperative precision, surgeon ergonomics, patient outcomes and reducing length of stay (LOS) [1, 2]. As robotic systems become more prevalent in operating rooms worldwide, caution should be taken to ensure that this technological advancement does not come at the expense of planetary health and sustainability for future generations. This commentary piece highlights, for surgeons and key stakeholders in robotic surgery, the environmental impact of current robotic technologies and the pressing need for responsible environmental stewardship. We do not advocate abandoning robotic surgery; rather, we call for accelerated innovation toward environmentally sustainable robotic platforms, and components that maintain clinical excellence while minimising ecological harm.

This article is intended as a commentary and perspective piece, informed by selected published literature and clinical experience. It is not a systematic or narrative review, nor is it intended to provide a comprehensive synthesis of the evidence. Rather, its purpose is to highlight key environmental challenges associated with robotic surgery and to outline practical priorities for clinicians, institutions, manufacturers, and policymakers.

## The environmental reality of robotic surgery

In Australia, the healthcare sector is a significant contributor to national greenhouse gas emissions, accounting for approximately 7% of the country’s total carbon footprint [3]. Within this, hospitals represent the largest share, and operating theatres are among the most resource-intensive environments due to continuous ventilation requirements, climate control, specialised equipment, and high consumables usage [4, 5]. A recent review of surgical sustainability further demonstrated that operating theatres contribute substantially to hospital waste production and energy use, with individual procedures exhibiting wide variation in carbon emissions depending on case duration, equipment selection, and disposable instrument utilization [6].

The environmental footprint of robotic surgery stems from multiple sources: energy-intensive console and robotic arm operation, substantial single-use instrument consumption, complex waste streams, and upstream manufacturing emissions. Recent life-cycle assessments reveal robotic systems draw approximately 3.5 kW during operation, generating roughly 4 kg CO_2_ per hour compared to 0.6 kW (1 kg CO_2_ per hour) for conventional laparoscopic equipment [7]. Per-procedure carbon emissions vary widely depending on surgical specialty and system boundaries, with reported ranges from 11.6 kg of CO_2_ (solid waste emissions only) for robotic colorectal procedures to 47.3 kg of CO_2_e (full life-cycle energy, materials and hospital stay) for prostatectomy per surgery [7–9]. Given the different methodological measurements between the surgeries, direct comparison across these procedures is unreliable. In general, per-procedure footprint is driven by operative duration, single-use instrument volume, energy device and stapler utilisation, and anaesthetic agent choice, not primarily by insufflation CO₂ gas [10], which represents a minor component of the overall footprint. Within colorectal surgery procedural complexity matters: robotic low anterior resection carries a higher footprint than simpler colonic resections owing to greater instrument burden and stapler use [11].

There is also a significant waste burden. Multi-specialty audits demonstrate that single-use items account for 93.9% of robotic case waste by weight, with components such as instrument arm drapes contributing up to 32.9% of total per-case waste [12]. Unlike traditional open surgery, which generates 7.1–22.7 kg CO_2_ per case in comparable procedures, robotic approaches concentrate disposable packaging and instrument-related waste which often end up incinerated or landfilled [9]. This reliance on single use items is often secondary to the difficulty of cleaning the robotic end effectors [13, 14], which presents significant challenges for validated sterilization [15]. While studies have investigated advanced sterilisation techniques [16], implementation remains hindered by fragmented international regulations and institutional policies [15]. Reliance is further driven by the economic model of robotic platforms; for instance, between 1999 and 2017, Intuitive Surgical Inc. derived 52% of its revenue ($1.64 billion USD) from instruments and accessories rather than initial system sales [17].

However, these figures tell an incomplete story. When life-cycle analyses incorporate the entire hospital episode, including reduced complications, shorter LOS, and faster patient recovery, some robotic procedures demonstrate lower total carbon footprints than their non-robotic laparoscopic counterparts [7]. This nuance highlights a critical point: the environmental impact of robotic surgery is not limited to the technology or operation itself, but rather to how we design, deploy, and dispose of these systems.

## The innovation imperative

The clinical benefits of robotic surgery are advancing, including enhanced three-dimensional visualisation, tremor filtration and enhanced ergonomics enable complex procedures through minimal incisions [18]. This translates to reduced blood loss, lower infection rates, and accelerated patient discharge [19]. Abandoning these advantages would be ethically untenable when millions of patients stand to benefit. Instead, as conscientious surgeons we must channel the same innovation that created robotic surgery toward making it environmentally responsible.

Several promising avenues already exist to improve sustainability. Manufacturers can alter current designs to move towards modular components, instigating serviceable architecture that extend device lifespan and facilitate component-level restoration rather than wholesale replacement [20]. The material selection of robotic components is a crucial determinant of environmental impact. Transitioning to recyclable composites allows for composite recycling using modern methods such as pyrolysis or solvolysis (thermal or chemical processing the material to regain the metal component for reuse) [21]. When designing instrument packaging, recycling and environmentally-friendly processes must be considered, implementing manufacturer take-back programs can dramatically reduce upstream emissions from repeated manufacturing [22]. Tjahyadi et al. extrapolated their waste results to the 2021/2022 RAS procedures performed in Australia, equating to 79,934 kg of waste [12], therefore, upstream design interventions offer substantial returns.

Hospitals and surgical teams possess immediate opportunities for impact reduction. Waste audits can identify “low-hanging fruit”, items opened but unused, excessive packaging, and opportunities for standardised preference card optimization [23, 24]. Systematic reviews of minimally invasive surgery (MIS) and sustainability demonstrate that reducing opened-but-unused instruments through evidence-based preference cards represents one of the most effective waste-reduction strategies [10]. Robotic surgery can reduce length of stay and with simple post-operative pathway changes, like encouraging telehealth appointments post-operatively, this can avoid 10–20 kg CO₂ per eliminated clinic visit [25].

A key component of MIS has been the advancement of surgical stapling technology, enabling clinicians to perform increasingly complex techniques such as intracorporeal anastomosis. However, surgical staplers contribute substantially to the environmental footprint of modern surgery [26]. Powered laparoscopic staplers are typically single-use devices and often rely on lithium batteries, resulting in a high volume of waste and resource consumption. While robotic surgery offers battery-less stapling systems, these devices are still predominantly single-use, designed for a single surgical procedure on a single patient before being discarded. Manufacturers frequently cite improved sterility as justification for this design; however, this approach does not align with environmentally sustainable surgical practice and contributes to the growing burden of healthcare-related waste [26]. A similar situation is seen with single use energy sealant devices.

## Toward a circular economy in surgical robotics

The transition to sustainable robotic surgery requires systems-level thinking that embraces circular economy principles. This means moving beyond incremental improvements toward fundamental redesign of how we manufacture, procure, use, and retire surgical robotic systems. As a pragmatic framework, we outline key stakeholder responsibilities that may help guide future sustainability efforts in robotic surgery (see Table 1).

**Table 1:** Stakeholder-specific contributions to reducing the environmental impact of robotic surgery

Stakeholder	Contributions
Manufacturers	• Modular product architectures, separating consumable and durable components • Transparent device-specific life-cycle assessment data • Refurbishment and take-back programs
Healthcare Institutions	• Incorporate supplier disclosure of life-cycle assessment (LCA) data alongside performance and cost metrics in procurement • Recycling pathways for non-hazardous plastics • Energy efficient operating room infrastructure • Routine waste audits • Implementation of mandatory preference-card optimisation
Surgical Teams	• Minimise open-but-unused items through closed-loop quality improvement initiatives • Encourage culture change in theatre allowing nursing staff to review unused opened items • Clinical champion validated instrument reprocessing programs • Drive demand for sustainable technologies • Model environmental stewardship • Telehealth reviews where practical for follow up
Policymakers and regulators	• Standardised LCA frameworks within device approval and post-approval surveillance • Mandate reporting of sustainability endpoints • Leverage frameworks similar to IDEAL [27] to enable transparent environmental benchmarking and incentivise competition on environmental performance

LCA, Life-cycle assessment, IDEAL; Idea, Development, Exploration, Assessment and Long-term monitoring

## Balancing patient benefit and planetary health

Some may argue that environmental considerations should never compromise patient care. This frames a false dichotomy. Emerging evidence suggests that when life-cycle analyses account for the complete hospital episode, including robotic surgery, it can deliver both superior clinical outcomes and competitive or lower environmental footprints compared to traditional approaches [7, 9]. The key lies in thoughtful implementation: selecting robotic surgery when clinical advantages are clear, optimising resource use during procedures, and systematically reducing waste and energy consumption.

Transparent evaluation is essential. Standardised sustainability targets in device assessment will empower clinicians and purchasers to select technologies that optimise both patient outcomes and environmental performance [27]. This transparency also drives manufacturer innovation [28]: when hospitals demand, and regulators require, environmental impact data, market forces align with sustainability goals.

Pursuing eco-friendly innovations in robotic surgery is not a compromise but an amplification of healthcare’s mission. Climate change poses profound threats to human health through heat-related illness, infectious disease spread [29], extreme weather events, water and food insecurity, conflicts over depleted resources [30] and healthcare system disruption [31]. By improving the sustainability of surgical technologies, we extend the health benefits of medical innovation across populations and generations.

## Conclusion

Robotic surgery represents one of modern medicine’s most transformative advances, and its trajectory should continue upward. Yet this trajectory must not compromise the environmental stewardship that is the responsibility of all surgeons. Taken together, the available literature and current practice experience suggest that targeted interventions in design, procurement, clinical practice, and waste management may reduce the environmental footprint of robotic surgery without undermining its clinical benefits.

The path forward requires collective action. Manufacturers must embrace circular design principles and transparent impact reporting. Hospitals must embed environmental criteria into procurement and operations. Surgical teams must champion resource optimisation and validated reprocessing. Regulators must standardise sustainability assessment frameworks. Together, these stakeholders can ensure that the next generation of surgical robotics delivers precision, safety, and environmental responsibility in equal measure.

Innovation brought us robotic surgery. Innovation will make it sustainable. The question is not whether we can afford to green robotic surgery, but whether we can afford not to.

## Data Availability

No datasets were generated or analysed during the current study.
